# Impact of enterovirus and other enteric pathogens on oral polio and rotavirus vaccine performance in Bangladeshi infants

**DOI:** 10.1016/j.vaccine.2016.04.080

**Published:** 2016-06-08

**Authors:** Mami Taniuchi, James A. Platts-Mills, Sharmin Begum, Md Jashim Uddin, Shihab U. Sobuz, Jie Liu, Beth D. Kirkpatrick, E. Ross Colgate, Marya P. Carmolli, Dorothy M. Dickson, Uma Nayak, Rashidul Haque, William A. Petri, Eric R. Houpt

**Affiliations:** a Division of Infectious Diseases and International Health, Department of Medicine, University of Virginia, Charlottesville 22908, USA; b Center for Vaccine Science and Parasitology Lab, International Centre for Diarrhoeal Disease Research, Bangladesh, Dhaka 1212, Bangladesh; c Vaccine Testing Center and Unit of Infectious Diseases, Department of Medicine, University of Vermont College of Medicine, Burlington, VT 05405, USA; d Center for Public Health Genomics, University of Virginia, Charlottesville 22908, USA

**Keywords:** OPV, oral polio vaccine, RV, rotavirus vaccine, NPEV, non-polio enterovirus, EV, enterovirus, PROVIDE, Performance of Rotavirus and Oral Polio Vaccines in Developing Countries, IPV, inactivated poliovirus vaccine, EAEC, enteroaggregative *E. coli*, EIEC, enteroinvasive *E. coli*, EPEC, enteropathogenic *E. coli*, ETEC, enterotoxigenic *E. coli*, STEC, Shiga-toxin producing *E. coli*, TAC, TaqMan Array Card, Oral polio vaccine, Rotavirus vaccine, Vaccine immunogenicity, Vaccine efficacy, Enteric infections, PCR

## Abstract

**Background:**

Oral polio vaccine (OPV) and rotavirus vaccine (RV) exhibit poorer performance in low-income settings compared to high-income settings. Prior studies have suggested an inhibitory effect of concurrent non-polio enterovirus (NPEV) infection, but the impact of other enteric infections has not been comprehensively evaluated.

**Methods:**

In urban Bangladesh, we tested stools for a broad range of enteric viruses, bacteria, parasites, and fungi by quantitative PCR from infants at weeks 6 and 10 of life, coincident with the first OPV and RV administration respectively, and examined the association between enteropathogen quantity and subsequent OPV serum neutralizing titers, serum rotavirus IgA, and rotavirus diarrhea.

**Results:**

*Campylobacter* and enterovirus (EV) quantity at the time of administration of the first dose of OPV was associated with lower OPV1-2 serum neutralizing titers, while enterovirus quantity was also associated with diminished rotavirus IgA (−0.08 change in log titer per tenfold increase in quantity; *P* = 0.037), failure to seroconvert (OR 0.78, 95% CI: 0.64–0.96; *P* = 0.022), and breakthrough rotavirus diarrhea (OR 1.34, 95% CI: 1.05–1.71; *P* = 0.020) after adjusting for potential confounders. These associations were not observed for Sabin strain poliovirus quantity.

**Conclusion:**

In this broad survey of enteropathogens and oral vaccine performance we find a particular association between EV carriage, particularly NPEV, and OPV immunogenicity and RV protection. Strategies to reduce EV infections may improve oral vaccine responses.

ClinicalTrials.gov Identifier: NCT01375647.

## Highlights

• Oral vaccines exhibit poor performance in low-income settings.
• Enterovirus and *Campylobacter* carriage are associated with lower OPV immunogenicity.
• Enterovirus carriage is associated with lower Rotarix immunogenicity and efficacy.

## 1 Introduction

The primary oral vaccines in use globally, OPV and RV, have poorer efficacy in infants in low-income settings compared to middle- to high-income countries [1], [2]. This observation was first noted with the introduction of OPV in the 1960s and 1970s [3], [4]. Subsequent studies have implicated several factors, including high maternal antibodies, concurrent breastfeeding, malnutrition, environmental enteropathy, prior or concurrent diarrhea, co-administration of other oral vaccines, or the presence of other enteric infections [5], [6], [7], [8], [9], [10]. Because these factors will co-exist in a given setting to varying degrees, documenting the predominance of a single factor such as enteric infections has been difficult. A recent meta-analysis of available studies, however, did demonstrate reduced odds of OPV seroconversion with concurrent non-polio enterovirus (NPEV) infection at the time of administration, particularly for type 1 OPV [11]. Notably, these studies were derived from the 1960s–1970s when the milieu of these other risk factors likely differed, and viral culture was standard practice to detect NPEV. Thus the impact of enteric infections on OPV and RV has not been re-visited in recent studies using modern molecular diagnostic techniques.

In the present-day Bangladeshi slum, exposure to enteric pathogens is ubiquitous from birth. Specifically, upon testing for a broad range of enteropathogens with molecular methods in birth cohorts, we have detected an average of more than 2 infections within the first month of life [12], particularly *Campylobacter* and *Escherichia coli* pathotypes. In this study we used molecular methods, which included all of the major viruses including enteroviruses, bacteria, protozoa, helminths, and fungi. We utilized a quantitative, singleplex, real-time PCR TaqMan Array Card platform that we have described previously [13], [14]. We analyzed stools collected prior to the first administration of OPV and RV at weeks 6 and 10 respectively to allow for a comprehensive, quantitative examination of concurrent enteric infections and subsequent oral vaccine performance.

## 2 Materials and methods

### 2.1 Study design and participants

This study was part of the “Performance of Rotavirus and Oral Polio Vaccines in Developing Countries (PROVIDE)” which followed infants from birth to 24 months of age in the urban slum area of Mirpur in Dhaka, Bangladesh to investigate the causes of underperformance of OPV and Rotarix in low-income settings [15]. In total 700 infants were recruited from November 2010 to August 2012. TAC testing was performed on subsets as indicated in Table 1. Infants were randomized to receive either the Bangladeshi Expanded Program on Immunization vaccine regimen of trivalent OPV (GlaxoSmithKline) at 6, 10, 14, and 38 weeks of age, versus substituted inactivated poliovirus vaccine (IPV; IMOVAX, Sanofi Pasteur) at 38 weeks. Infants in both arms were independently randomized to receive Rotarix vaccine (GlaxoSmithKline) or not at 10 and 17 weeks of age. Stool samples were collected either at the clinic or the participant's home before vaccinations and delivered to our lab while maintaining cold chain within 6 h of collection and stored in −80 °C until further processing. We tested by TAC pre-6 week vaccination stools from a subset of 339 infants (166 female, 173 male), who were chosen randomly from the cohort. From this subset, we additionally tested stools collected at week 10 prior to the first dose of Rotarix from the 159 children who received rotavirus vaccine per protocol. Infants were followed via twice-weekly home visits from week 18–52 for incident diarrhea and diarrheal stool specimens were collected and tested for rotavirus by ELISA (ProSpecT, Oxoid, UK). Informed consent was obtained from all participants’ parents or guardians. This study was approved by the Institutional Review Committee and the Institutional Ethical Committee at the International Centre for Diarrhoeal Disease Research, Bangladesh (icddr,b) and the Institutional Review Boards at the University of Vermont and the University of Virginia.

### 2.2 Vaccine antibody titers

Sera obtained from infants at 6 and 18 weeks of age were shipped on dry ice from Bangladesh to the Centers for Disease Control in Atlanta, USA for neutralizing antibody assays for poliovirus serotype 1, 2, and 3 (log 2 based titer) which were performed according to World Health Organization methods [16]. Rotavirus plasma IgA was measured as described previously [17]. OPV seroconversion was defined as both a week 18 neutralizing antibody titer greater than 2.83 (log 2 based titer) and a change in the log titer between weeks 6 and 18 of at least 3 (to account for decay in maternally-transferred antibody from week 6 to week 18 with a half-life of 4 weeks). Rotavirus serconversion was defined as both a serum rotavirus IgA greater than 20 U/mL at week 18 and a titer of less than 20 U/mL at week 6.

### 2.3 RNA and DNA extraction from stool

RNA was extracted from stool using a slightly modified protocol of the QIAamp Viral RNA Mini Kit (Qiagen, Gaithersburg, MD) [18]. Briefly, 100–200 mg of stool specimen was added to 1 ml of 0.89% NaCl solution in a 2 ml screw cap tube and vortexed. The suspension was centrifuged at 4000 × *g* for 20 min then 140 μl of the supernatant was added to 560 μl of Buffer AVL containing carrier RNA and 1 μl of bacteriophage MS2 (approximately 2 × 10^6^ copies, ATCC 15597B1; American Type Culture Collection, Manassas, VA) per sample to serve as an extraction and amplification control. The suspension was mixed thoroughly then incubated at room temperature for 10 min. After incubation, 560 μl of ethanol was added then mixed by pulse-vortexing for 15 s. An aliquot of 630 μl was applied to the QIAamp Mini column and then followed the manufacturer's recommended protocol supplied with the kit. The RNA was stored with 60 μl of RNA Storage Solution (Ambion, Foster City, CA) in −80 °C until testing. DNA was extracted using a modified QIAamp DNA Stool Mini Kit protocol (Qiagen, Gaithersburg, MD) [19]. Briefly, 1.4 ml of the lysis buffer ASL with extrinsic control, phocine herpes virus (PhHV), was added to the 200 mg of stool, then the suspension was pretreated by bead beating with glass beads (Sigma–Aldrich, St. Louis, MO) for 2 min followed by incubation for 7 min at 95 °C before proceeding with the extraction as per manufacturer's protocol. DNA was stored in −80 °C until testing.

### 2.4 Molecular diagnostics

Custom-developed TaqMan Array Cards (TAC) were utilized for detection of infections in stool. Briefly, RNA and DNA were combined and tested using the methodology, master mix, and cycling conditions previously described on a Viia7 platform (Life Technologies, South San Francisco, CA) [13]. Validation of the platform has been described [14]. We tested for alphabetically, the following infections using those previously described assays with additions as specified: adenovirus 40/41 [20], *Aeromonas* spp. [21], *Ancylostoma duodenale* [22], *Ascaris lumbricoides*, astrovirus, *Bacteroides fragilis* [23], *Campylobacter* spp. [24], *Clostridium difficile*, *Cryptosporidium hominus*/*parvum* [25], *Cyclospora cayetanensis* [26], *Cystoisospora belli* [27], *Encephalitozoon intestinalis* [28], *Entamoeba histolytica*, *Enterocytozoon bieneusi* [28], enteroaggregative *E. coli* (EAEC), enteroinvasive *E. coli* (EIEC)/*Shigella*, enteropathogenic *E. coli* (EPEC), enterotoxigenic *E.coli* (ETEC), pan-enterovirus [29], *Giardia lamblia*, *Helicobacter pylori*, *Mycobacterium tuberculosis* [30], *Necator americanus* [22], norovirus GI [31], norovirus GII, rotavirus, *Salmonella* spp., sapovirus, Shiga-toxin producing *E. coli* (STEC), *Strongyloides stercoralis* [32]), *Trichuris trichiura* and *Vibrio cholerae* [21]. Virulence genes were used to define the *E. coli* pathotypes as follows: *aaiC* and/or *aatA* for EAEC, *ipaH* for EIEC/*Shigella*, ST and/or LT for ETEC, *eae* with or without *bfpA* for EPEC, and *stx1* and/or *stx2* for STEC. Amplification after threshold cycle (Ct) above 35 was considered negative. In addition, all available 10 week stool from children who received RV per protocol and were followed until at least one year of age were tested by the cognate pan-EV RT-qPCR on plates. Briefly, 0.8 μl Ag-Path One-Step RT enzyme (Life Technologies, South San Francisco, CA), 10 μl Ag-Path 2× buffer, 7.2 μl nuclease free water, 1 μl of enterovirus assay (primer and probe mix) and 1 μl of RNA was tested in 20 μl reaction and run on a CFX cycler (Bio-Rad, Ventura, CA) with cycling conditions: 45 °C for 20 min, 95 °C for 10 min, and 45 cycles of 95 °C for 15 s and 60 °C for 1 min. To further describe EV infections in these stools, we utilized a multiplex RT-qPCR assay to identify Sabin strain polioviruses [33].

### 2.5 Viral culture

Stools from children prior to the 14 week OPV administration underwent viral culture for polio and NPEV according to the methodologies of the World Health Organization's Polio laboratory manual (4th edition) [16]. Briefly, stool cultures that were positive on RD cell lines but negative on LB20 cell lines were identified as NPEV.

### 2.6 Statistical analysis

To estimate associations between pathogen quantity and vaccine titers, seroconversion, and breakthrough rotavirus diarrhea, linear regression was used for continuous outcomes and logistic regression for binary outcomes. In all cases, antibody titers were log transformed. For the linear regression analysis of OPV and rotavirus titers, baseline titers obtained prior to OPV administration at week 6 were included as covariates. For all analyses, pathogen burden was defined as the number of non-enterovirus enteropathogens detected in a stool. Additional covariates, including gender, income, treatment of drinking water, weight-for-age *Z* score at 10 weeks, number of episodes of diarrhea through 6 weeks of age, weeks of exclusive breastfeeding through week 18, and serum zinc level at 18 weeks were included in the adjusted regression models based on model fit as determined by the Akaike Information Criterion (AIC). If the AIC was similar between candidate models, covariates were retained. Other covariates considered but not included were Vitamin D level at 18 weeks, maternal education, length-for-age *Z* score at 10 weeks, and retinol binding protein at 18 weeks. The Wilcoxon rank sum test was used to compare antibody titers and pathogen burden between groups. All *P*-values were considered statistically significant at a level of 0.05. All analyses were performed in R 3.2.2 (R Foundation for Statistical Computing, Vienna, Austria [2015]).

## 3 Results

### 3.1 Enteric infections at the time of vaccination

**Fig. 1:**
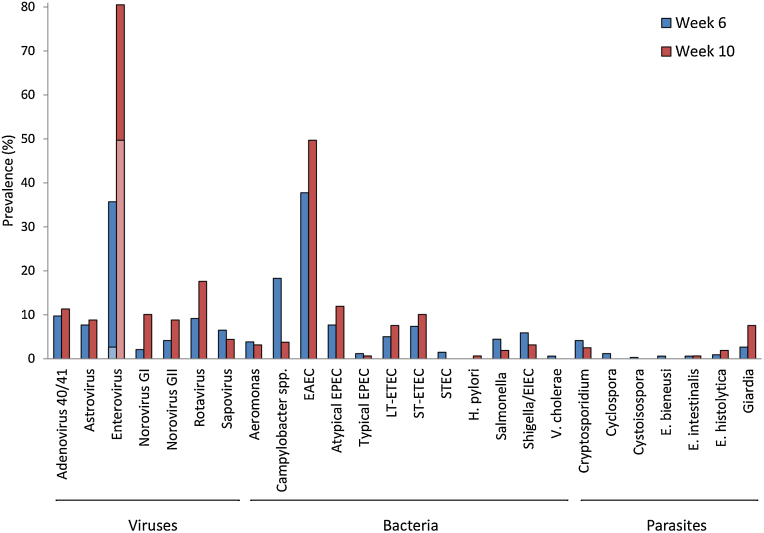
Prevalence of enteric infections detected by quantitative PCR in week 6 (*n* = 339) and 10 (*n* = 159) pre-vaccination stool specimens. Pre-vaccination stool specimens were collected at the time points indicated and assayed for enteropathogens by TAC. All tested helminths and *M. tuberculosis* were exceedingly rare (0–3%) and are not shown. All infections were tested by TAC except OPV; OPV prevalence is overlaid on enterovirus prevalence with light blue for week 6 and light red for week 10).

TAC was performed on stool samples from 339 randomly selected infants at week 6 of life prior to the first OPV administration and, for the 159 of these infants who received RV per protocol, at week 10 prior to the first RV administration. The mean number of pathogens detected in the pre-vaccination stool specimens was 1.7 ± 1.4 pathogens at week 6 and 2.4 ± 1.4 pathogens at week 10 (*P* < 0.001). At least 1 infection was detected in 82.0% of week 6 and 96.9% of week 10 stools, and multiple infections were abundant (48.7% and 73.0% had 2 or more pathogens at the two time points, respectively). The specific pathogens are shown in Fig. 1.

### 3.2 Specific enteric infections and their association with OPV immunogenicity

**Fig. 2:**
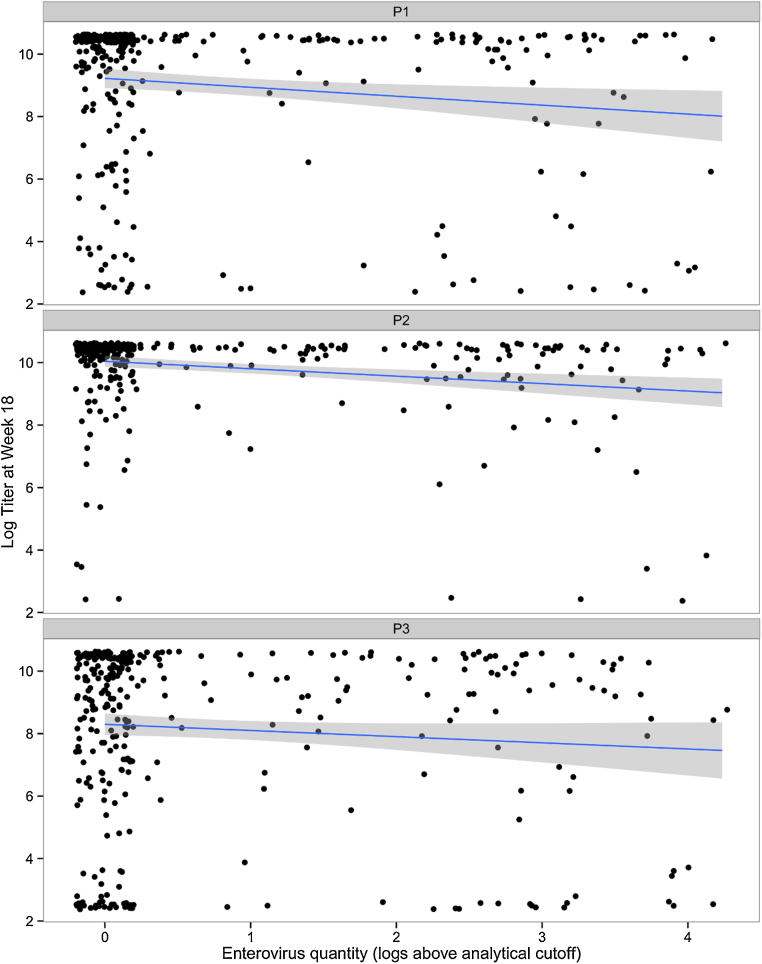
Association between EV quantity and serum neutralizing antibody titers to serotype P1, P2, and P3. Enterovirus-negative samples are jittered on the *x*-axis.

An association was observed between the number of pathogens detected at the time of the first OPV administration (6 weeks) and a reduced OPV serum neutralizing titer (18 weeks) for all 3 types, however after adjusting for potential confounders, statistical significance was seen only for P2 (Table 2). That said, in the subset tested by TAC, seroconversion to P2 was high (95% vs. 86% and 83% for P1 and P3). Since most individual infections were rare at these early time points, and to avoid spurious associations, we examined the association between those pathogens with at least a 10% prevalence (*Campylobacter*, EAEC, EV) and OPV immunogenicity using multivariable linear and logistic regression. Pathogens significantly associated with lower OPV serum neutralizing titer were *Campylobacter* and EV for P1 and P2 (Table 2). Fig. 2 illustrates these data by showing EV quantity versus OPV1-3 serum neutralizing titer.

All pan-EV TAC positive stools (121/339 (35.7%)) were further tested for OPV1-3 by qRT-PCR, of which only 8/121 (6.6%) were positive, suggesting this EV effect on OPV was due to NPEV and not circulating Sabin viruses. The association between OPV serum neutralizing antibody and NPEV carriage was also seen by stool culture prior to OPV administration at week 14 (mean P1 titer 7.83 ± 3.17 in NPEV positive vs. 9.14 ± 2.41 in NPEV negative, *P* = 0.021; mean P2 titer 9.61 ± 1.75 vs. 9.89 ± 1.40, *P* = 0.066; mean P3 titer 6.79 ± 3.12 vs. 8.32 ± 2.70, *P* < 0.001).

### 3.3 Specific enteric infections and their association with Rotarix immunogenicity and efficacy

**Supplementary Fig. 1:**
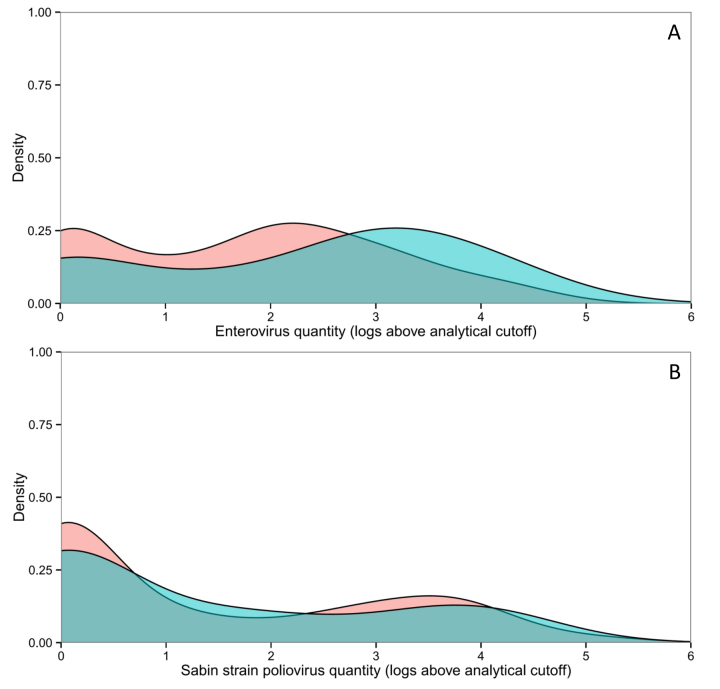
Association between EV burden and Rotarix efficacy. Density plots of fecal EV (A) and Sabin strain poliovirus (B) quantity at 10 weeks in children with (blue, *n* = 45) and without (red, *n* = 232) rotavirus-positive diarrhea between weeks 18 and 52. For each color, the area under the curve for any range of pathogen quantities represents the probability that the pathogen quantity falls in that range.

We then determined the association between enteric infections at the time of the first dose of RV at week 10 and serum rotavirus IgA measured post-RV at week 18. In the subset of 159 infants tested by TAC at week 6 who received RV per protocol, TAC was also performed on week 10 stools, and EV quantity was again associated with reduced rotavirus IgA titer, whereas no other enteric infection exhibited a significant association (Table 3). To further investigate the association between enterovirus and rotavirus vaccine performance, we tested available week 10 stools from the remaining infants who received RV per protocol (*n* = 277) for EV by PCR. These infants were followed from weeks 18–52 of life to identify episodes of rotavirus diarrhea confirmed by enzyme immunoassay. 45 of 277 had at least one episode of rotavirus diarrhea during this time period. In the adjusted analysis, EV infection at week 10 was associated with increased odds of breakthrough rotavirus diarrhea (Table 4, OR 1.34 per tenfold increase in EV quantity; *P* = 0.020; Supplemental Fig. 1A). To better understand the identity of these EV, we tested each EV PCR-positive stool by PCR for Sabin strain poliovirus. EV was detected in 221 of 277 stools, and 150 of 221 (67.9%) were positive for OPV1-3, presumably reflecting shedding of the week 6 OPV dose. However, OPV1-3 quantity was not significantly associated with impaired RV immunogenicity (Table 4) or efficacy (Table 4 and Supplemental Fig. 1B), suggesting that the association between EV and vaccine performance was specific to NPEV. Finally, since EV infection may simply be a marker for other risk factors associated with breakthrough rotavirus diarrhea, we adjusted for possible risk factors for rotavirus diarrhea identified in the primary analysis of RV effectiveness from the PROVIDE study in children who received RV per protocol, including micronutrients, nutritional status, breastfeeding and water treatment [34], [35]. Following adjustment for confounders, EV quantity in stool was independently associated with a lower rotavirus serum IgA titer, failure to seroconvert, and breakthrough rotavirus diarrhea in the first year of life (Table 4), while no association was seen for Sabin strain poliovirus quantity.

## 4 Discussion

The major finding of this paper is not only the confirmation of the deleterious role of NPEV on OPV and RV response, but the seemingly selective role of NPEV versus other known enteropathogens, all of which were interrogated with highly sensitive molecular diagnostics. The only exception would appear to be *Campylobacter* species, which was also associated with impaired OPV immunogenicity. An important feature of this work was the quantitative analysis between enteric infection and oral vaccine responses, something that has not been done in historical studies because qPCR for such a broad panel of infections was not previously possible.

It has long been suspected that enterovirus infection could interfere with oral polio vaccine performance. It has been postulated that concurrent NPEV infection may interfere with Sabin vaccine uptake because receptors may be conformation ally nearby on the enterocyte, or NPEV may induce antiviral immunity which then prevents Sabin viruses from establishing an infection [11]. We in fact found an association of concurrent EV on OPV serum neutralizing response, particularly for P1. Additionally, we observed an association between *Campylobacter* quantity in stool prior to the first OPV administration and subsequent diminished OPV1-3 titers. Few studies have examined the role of *Campylobacter* on OPV. In the one relevant study of Mexican infants, *Campylobacter* culture positivity was not associated with a diminished response to the first OPV dose, but did contribute to an effect on OPV1 and 2 at the second dose [7]. We have previously shown that *Campylobacter* PCR is vastly more sensitive than culture, which may explain some of this difference [36]. Whether *Campylobacter* infection is a marker for other risk factors or directly causal to the impaired OPV response requires additional study, such as through antibiotic trials. We would note that the *Campylobacter* effect was most significant for the P2 titer, which is the least problematic Sabin type, and impaired P2 response also correlated generically with an increased number of enteric infections. Therefore the P2 immunogenicity lesion noted in this study may reflect a small number of infants with generally compromised immunity (to OPV2, *Campylobacter*, and other infections).

Relatively less is understood about concurrent enteric infection on RV response, outside of direct OPV-RV co-administration studies. These have been performed in a few countries, including Bangladesh, with small sample sizes and various vaccine regimens, but have generally shown a modestly reduced serum rotavirus IgA titer or seroconversion rate after co-administration [5], [8], [37]. This can generally be overcome with multiple RV administrations thus the clinical significance of this observation has been uncertain. The role of enteric infections other than OPV on RV has been even less studied. In vitro, however, competition studies with EV and rotavirus have revealed preferential replication of EV, suggesting such infections could impact RV response [38]. In this context, our finding of the negative association between EV infection and rotavirus IgA titer is novel, particularly since this association did not track with OPV infection. Moreover, this NPEV effect on RV was also seen clinically, with increases in breakthrough RV diarrhea in those with higher EV (but not OPV) quantity at the time of the first RV. The deleterious effect of EV on breakthrough diarrhea held up against multivariable regression for potential confounding risk factors for RV diarrhea, such as poverty, malnutrition, and water quality. Evidence for a negative clinical impact on RV efficacy by concurrent enteric infections has eluded previous studies, probably because these have been performed in high or middle income countries where concurrent infections and RV transmission is low while immunogenicity is high [39].

As for possible interventions, the effect size of EV quantity on immunogenicity and RV efficacy did appear substantial compared with other risk factors, so studies to evaluate EV prevention measures should be pursued. Unfortunately the genus is comprised of 12 species and 5 serotypes (coxsackievirus, echovirus, enterovirus, rhinovirus, and poliovirus). These are ubiquitous, among the most common viruses infecting humans, and spread person to person via direct and indirect routes. Hygienic measures such as hand-washing can prevent acquisition of certain strains [40], and it will be valuable to evaluate if particular water, sanitation, and hygiene interventions are effective in reducing EV burden and thus could be expected to impact oral vaccine performance.

There were limitations to this study. The study was performed in one single site, where the exact profile of risk factors for impaired oral vaccine response may not be generalizable. The sample size was modest, we could not perform TAC on all stools at all time points, and thus the power to observe effects of rarer pathogens was low. However, associations between rare pathogens and impaired oral vaccine performance would be unlikely to have significant public health implications. Detection of nucleic acid does not necessarily denote replicating organism, however higher quantity infections do correlate with culture positivity [14], [33]. Another limitation is that we did not have serotype-specific polio neutralizing antibody titers after each OPV administration nor rotavirus IgA titers after vaccination at weeks 10 and 17, which would have enabled per-dosage examinations of titers. Because the pan-EV qPCR assay will detect OPV, the identified associations with NPEV are based on the subtractive logic of an EV association without an OPV association. Direct discrimination of the panoply of enteroviruses by next generation sequencing will be required to further dissect and confirm this. In addition, the mechanisms by which NPEV impair the effectiveness of oral vaccines deserves further evaluation, particularly in studies which can compare the impact of NPEV in both vaccinated and unvaccinated children exposed to RV.

## 5 Conclusions

In summary, our quantitative analysis of a broad spectrum of enteric pathogens at the time of the first OPV administration shows that EV and *Campylobacter* quantity was associated with lower immunogenicity. For the first time, an association was also seen between EV carriage and both Rotarix immunogenicity and efficacy. Strategies to reduce EV transmission could complement other approaches to improving oral vaccine performance in these settings.

## Figures and Tables

**Table 1:** Schematic of this sub-study within PROVIDE. Infants underwent stool enteropathogen testing and immunologic and efficacy follow-up at the time points indicated.

Week of life	6	10	14	17	18	18–52
Vaccination	tOPV	tOPV	tOPV			
		Rotarix		Rotarix		
OPV sub-study	TAC of stool prior to tOPV (*n* = 339)		Culture of stool for EV prior to tOPV		Outcomes: OPV1-3 serum neutralizing titer and seroconversion	
Rotarix sub-study		TAC of stool prior to Rotarix (*n* = 159); qRT-PCR for pan-EV and Sabin strains (*n* = 277)			Outcomes: Serum rotavirus IgA and seroconversion	Outcome: Rotavirus diarrhea

**Table 2:** Association between pathogen burden as well as specific pathogen quantities and the week 18 OPV serum neutralizing titer in stools collected prior to first OPV dose (*n* = 339). For each serotype, results were adjusted for gender, income, treatment of drinking water, weight-for-age *Z* score at week 10, and number of diarrheal episodes through 6 weeks of age using multivariable linear regression.

Serotype (mean titer ± SD)	Pathogen	Change in titer				Seroconversion			
		Crude^a^		Adjusted^b^		Crude^a^		Adjusted^b^	
		Change in log polio titer	*P* value	Change in log polio titer^b^	*P* value	Odds ratio (95% CI)	*P* value	Odds ratio (95% CI)	*P* value
P1 (9.01 ± 2.52)	Total pathogen burden^c^	−0.15	0.111	−0.19	0.055	0.90 (0.73–1.10)	0.296	0.84 (0.67–1.05)	0.134
	*Campylobacter* spp.	−0.27	0.044	−0.29	0.036	0.82 (0.63–1.06)	0.133	0.81 (0.62–1.05)	0.114
	Enterovirus	−0.29	0.009	−0.28	0.014	0.80 (0.64–1.02)	0.069	0.81 (0.63–1.04)	0.097
	EAEC	0.14	0.069	0.15	0.060	1.31 (1.02–1.67)	0.032	1.33 (1.03–1.72)	0.031

P2 (9.87 ± 1.44)	Total pathogen burden^c^	−0.21	<0.001	−0.18	0.001	0.61 (0.45–0.81)	<0.001	0.67 (0.49–0.91)	0.011
	*Campylobacter* spp.	−0.23	0.003	−0.20	0.011	0.71 (0.50–1.01)	0.060	0.77 (0.52–1.16)	0.211
	Enterovirus	−0.23	<0.001	−0.22	<0.001	0.51 (0.36–0.74)	<0.001	0.53 (0.36–0.77)	<0.001
	EAEC	−0.07	0.095	−0.05	0.298	0.75 (0.58–0.96)	0.021	0.79 (0.61–1.03)	0.083

P3 (8.15 ± 2.79)	Total pathogen burden^c^	−0.21	0.046	−0.17	0.113	0.84 (0.70–1.02)	0.073	0.88 (0.72–1.08)	0.227
	*Campylobacter* spp.	−0.30	0.043	−0.22	0.145	0.78 (0.62–0.99)	0.040	0.83 (0.65–1.07)	0.143
	Enterovirus	−0.19	0.122	−0.14	0.264	0.83 (0.67–1.04)	0.100	0.87 (0.69–1.09)	0.226
	EAEC	−0.04	0.624	−0.03	0.723	0.95 (0.81–1.11)	0.539	0.98 (0.83–1.16)	0.811

a Estimate is per additional pathogen for total pathogen burden and per tenfold increase in pathogen quantity for individual pathogens, and is adjusted for the week 6 OPV serum neutralizing titer.

b Additionally adjusted for gender, income, treatment of drinking water, weight-for-age *Z* score at 10 weeks, number of episodes of diarrhea through 6 weeks of age, weeks of exclusive breastfeeding through week 18, and serum zinc level at 18 weeks.

c Total pathogen burden was defined as the sum total of non-enterovirus pathogens detected at analytical cutoff of Ct < 35.

**Table 3:** Association between specific pathogens in stools collected prior to first Rotarix dose and serum anti-Rotavirus IgA titer at 18 weeks by linear regression (*n* = 159).

Pathogen	Serum IgA titer		Seroconversion	
	Change in log rotavirus serum IgA titer^a^	*P* value	Odds ratio (95% CI)^a^	*P* value
Adenovirus 40/41	0.06	0.698	1.06 (0.90–1.24)	0.486
Enterovirus	−0.15	<0.001	0.93 (0.89–0.98)	0.004
Norovirus GI	−0.02	0.741	0.96 (0.89–1.04)	0.322
Rotavirus	0.15	0.121	1.10 (0.99–1.22)	0.086
EAEC	−0.06	0.108	0.98 (0.94–1.02)	0.295
Atypical EPEC	−0.05	0.563	0.95 (0.87–1.04)	0.266
ST-ETEC	0.02	0.836	1.03 (0.91–1.16)	0.665

a Estimate is per tenfold increase in pathogen quantity and is adjusted for the week 6 rotavirus serum IgA titer.

**Table 4:** Association between enterovirus and Sabin strain poliovirus quantity and rotavirus IgA titer at 18 weeks, rotavirus seroconversion at 18 weeks, and breakthrough rotavirus diarrhea between weeks 18–52 in children who received Rotarix per protocol (*n* = 277).

	Serum IgA titer at 18 weeks				Seroconversion at 18 weeks				Rotavirus diarrhea between weeks 18–52			
	Crude^a^		Adjusted^b^		Crude^a^		Adjusted^b^		Crude^a^		Adjusted^b^	
	Change in log rotavirus titer^c^	*P* value	Change in log rotavirus titer^c^	*P* value	Odds Ratio (95% CI)^c^	*P* value	Odds Ratio (95% CI)^c^	*P* value	Odds Ratio (95% CI)^c^	*P* value	Odds Ratio (95% CI)^c^	*P* value
Enterovirus quantity in stool, 10 weeks	−0.10	0.010	−0.08	0.037	0.90 (0.85–0.97)	0.007	0.78 (0.64–0.96)	0.022	1.31 (1.03–1.66)	0.026	1.34 (1.05–1.71)	0.020
Sabin strain poliovirus quantity in stool, 10 weeks^c^	−0.04	0.195	−0.02	0.474	0.97 (0.82–1.15)	0.765	0.99 (0.83–1.18)	0.904	0.98 (0.80–1.19)	0.823	0.99 (0.80–1.22)	0.992

a Adjusted for the serum rotavirus IgA titer at week 6.

b Additionally adjusted for gender, income, treatment of drinking water, weight-for-age *Z* score at 10 weeks, number of episodes of diarrhea through 10 weeks of age, weeks of exclusive breastfeeding through week 18, and serum zinc level at 18 weeks.

c Estimate is per tenfold increase in pathogen quantity.
