# Supplementary material for: Impact of enterovirus and other enteric pathogens on oral polio and rotavirus vaccine performance in Bangladeshi infants
Source: Vaccine. 2016 Jun 8;34(27):3068–75. doi: 10.1016/j.vaccine.2016.04.080 (PMC4912219; doi:10.1016/j.vaccine.2016.04.080)
Supplement: Supplementary file 1 [file mmc1.docx]

Figure 1: CONSORT Flow Diagram

1048 Screened

347 Ineligible

1 Refused consent

700 Randomized

350 RV1 arm

350 No RV1 arm

16 Consent withdrawn

3 Moved away

3 Infant death

16 Consent withdrawn

10 Moved away

2 Missed visit

9 Visit outside window

365 days surveillance

Week 17

RV1 dose 2

Week 10

RV1 dose 1

320

292

301

298

331

310

1 Infant death

7 Consent withdrawn

3 Moved away

8 Lost to follow up

6 Consent withdrawn

2 Moved away

3 Lost to follow up

1 Infant death

4 Consent withdrawn

1 Moved away

1 Consent withdrawn

4 Missed visit

7 Visit outside window

Table 1: Baseline characteristics by randomization arm

|  | Randomization Arm | |
| --- | --- | --- |
|  | **RV1 arm (n=350)** | **No RV1 arm (n=350)** |
| *Child features* | | |
| Gender, male n (%) | 182 (52.0) | 186 (53.1) |
| Median age at enrollment (days) | 5 (1 – 7) | 5 (1 – 7) |
| Weight at enrollment (kg) | 2.7 (1.7 – 4.1) | 2.8 (1.9 – 4.0) |
| Length at enrollment (cm) | 48.5 (43.1 – 55.4) | 48.8 (44.5 – 54.6) |
| Height-for-age z score at enrollment | -0.96 (-3.67 – 2.88) | -0.85 (-3.07 – 1.83) |
| Weight-for-age z score at enrollment | -1.28 (-4.00 – 1.24) | -1.26 (-3.33 – 1.00) |
| Excl. Breastfeeding at 18 weeks, n (%)^b^ | 154 (50.0) | 161 (53.5) |
| Home birth, n (%) | 100 (28.6) | 81 (23.1) |
| *Maternal features* | | |
| Age at enrollment (years) | 24 (18 – 40) | 24 (18 – 41) |
| Vaginal delivery, n (%) | 277 (79.1) | 263 (75.1) |
| Height (cm)^c^ | 150 (137– 187) | 150 (134 – 167) |
| Post-partum weight (kg)^c^ | 48.0 (30.2 – 80.0) | 47.0 (30.0 – 77.0) |
| Other children ≤5 years old in home | 92 (26.3) | 96 (27.4) |
| Mother illiterate | 107 (30.6) | 95 (27.1) |
| *Household and Socioeconomic features* |  |  |
| Total monthly income, 1000Taka | 10 (3 – 77) | 10 (3 – 70) |
| Piped municipal water, n (%) | 339 (96.9) | 339 (96.9) |
| Toilet or septic tank, n (%) | 195 (55.7) | 172 (49.1) |
| One-room home, n (%) | 251 (71.7) | 256 (73.1) |
| Household members | 5 (1 – 16) | 4 (2 – 18) |

Figures are n (%) or Median (range)

^a^ N=190 RV1 arm, N=191 No RV1 arm

^b^ N=308 RV1 arm, N=301 No RV1 arm

^c^ N=330 RV1 arm, N=339 No RV1 arm

Table 2: Incidence of rotavirus diarrhea in PROVIDE compared with other cohorts.

|  | **Rotavirus Diarrhea** | | | **Severe Rotavirus Diarrhea** | | |
| --- | --- | --- | --- | --- | --- | --- |
|  | Cases (N) | Person Years | Incidence ^a^ | Cases (N) | Person Years | Incidence ^a^ |
| PROVIDE (urban, unvaccinated) | 121 | 315.9 | 38.3 | 41 | 315.9 | 13 |
| Rural Bangladesh and Vietnam [6] | 109 | 1143.4 | 9.5 | 71 | 1156.9 | 6.1 |
| Sub-Saharan Africa [7] | 294 | 2556.3 | 11.5 | 129 | 2585.9 | 5.0 |
| South Africa and Malawi [10] | NA | NA | NA | 70 | NA | 8.0 |

^a^ Incidence per 100 person years

NA means Not Available

Table 3: Rotavirus diarrhea incidence and vaccine efficacy, Intention-to-treat (ITT) and Per-Protocol (PP) analyses.

|  | **All Subjects %**  **(95% CI)** | **RV1 arm** %  **(95% CI)** | **No RV1 arm** %  **(95% CI)** | **Risk Difference %**  **(95% CI)** | **P-value** | **RR**  **(95% CI)** | **Efficacy %**  **(95% CI)** |
| --- | --- | --- | --- | --- | --- | --- | --- |
| **Year 1 ITT Analysis, N=700** | | | | | | | |
| Rotavirus diarrhea | 25.8  (22.7 – 29.2) | **19.1**  (15.3 – 23.6) | **32.6**  (27.8 -37.6) | **13.4**  (7.0 – 19.8) | 4.0 × 10^–5^ | **1.70**  (1.31 – 2.21) | **41.2**  (23.6 – 54.8) |
| Severe Rotavirus diarrhea | 7.6  (5.8 – 9.8) | **4.0**  (2.4 – 6.6) | **11.1**  (8.2 – 14.9) | **7.1**  (3.2 – 11.2) | 3.0 × 10^–4^ | **2.78**  (1.54 – 5.02) | **64.1**  (35.1 – 80.1) |
| All cause diarrhea | 85.7  (82.9 – 88.1) | 85.1  (81.0 – 88.5) | 86.3  (82.3 – 89.5) | 1.1  (-4.1 – 6.4) | 0.66 | 1.01  (0.95 – 1.08) | 1.3  (-4.8 – 7.1) |
| Severe all cause diarrhea | 33.7  (30.3 – 37.3) | 31.4  (26.8 – 36.5) | 36.0  (31.1 – 41.2) | 4.6  (-2.4 – 11.5) | 0.20 | 1.14  (0.93 – 1.41) | 12.7  (-7.5 – 29.1) |
| **Post-vaccination Per Protocol Analysis, N=593** | | | | | | | |
| Rotavirus diarrhea | 25.6  (22.3 – 29.3) | **16.8**  (12.9 – 21.5) | **34.2**  (29.1 -39.8) | **17.4**  (10.5 – 24.2) | 6.6 × 10^–7^ | **2.04**  (1.51 – 2.75) | **51.0**  (33.8 –63.7) |
| Severe Rotavirus diarrhea | 7.4  (5.6 – 9.8) | **3.1**  (1.6 – 5.8) | **11.6**  (8.5 – 15.7) | **8.5**  (4.4 – 12.9) | 5.0 × 10^–5^ | **3.77**  (1.85 – 7.71) | **73.5**  (45.8 – 87.0) |

Figure 2: Month of age at first confirmed episode of rotavirus diarrhea

Table 4: Evaluation of variables associated with risk of rotavirus diarrhea: Univariate logistic regression

| **Variable** | **Odds Ratio (95% CI)** | ***P*-value** |
| --- | --- | --- |
| **Rotavirus Diarrhea in year 1** | | |
| No RV1 arm (control) | 2.34 (1.62 – 3.37) | **5 × 10^–6^** |
| Zinc, 18 weeks (x 100ug/L) | 0.82 (0.72 – 0.94) | 0.003 |
| Duration excl. breast feeding, 18 weeks | 0.97 (0.94 – 0.99) | 0.017 |
| Absence of water treatment | 1.47 (1.02 – 2.10) | 0.037 |
| Vitamin D, 18 weeks | 1.01 (1.00 – 1.02) | 0.045 |
| Sex of child (male) | 1.41 (0.99 – 2.02) | 0.058 |
| WAZ, 10 weeks | 0.83 (0.69 – 1.01) | 0.058 |
| Monthly income (1000 Taka) | 1.01 (0.99 – 1.03) | 0.155 |
| Mother education (None) | 0.85 (0.57 – 1.26) | 0.421 |
| HAZ, 10 weeks | 0.94 (0.77 – 1.14) | 0.520 |
| Rotavirus IgA seronegative, 18 weeks | 1.09 (0.73 – 1.65) | 0.663 |
| Retinol binding protein, 18 weeks (ng/L) | 1.00 (0.99 – 1.01) | 0.970 |
| **Severe Rotavirus Diarrhea in year 1** | | |
| No RV1 arm (control) | 3.18 (1.68 – 5.99) | **1.5 × 10^–4^** |
| Zinc, 18 weeks (x 100ug/L) | 0.93 (0.75 – 1.14) | 0.463 |
| Duration excl. breast feeding, 18 weeks | 0.97 (0.93 – 1.01) | 0.164 |
| Absence of water treatment | 1.30 (0.74 – 2.30) | 0.366 |
| Monthly income (1000 Taka) | 1.03 (1.00 – 1.05) | 0.031 |
| Gender (Male) | 1.03 (0.59 – 1.82) | 0.904 |
| WAZ, 10 weeks | 0.99 (0.73 – 1.34) | 0.957 |

*P*-values from Likelihood Ratio Test

Table 5: Rotavirus specific plasma IgA: Children seropositive and seroconverted at weeks 6 (pre-vaccination) and 18 (post-vaccination).

| **Group** | **Time point** | **N** | **Seropositive^a^** | | | **N** | **Seroconversion^b^** | | |
| --- | --- | --- | --- | --- | --- | --- | --- | --- | --- |
|  |  |  | **n** | **%** | **95% CI** |  | **n** | **%** | **95% CI** |
| All Children | Week 6 | 590 | 27 | 4.6 | 2.9 – 6.3 | -- | -- | -- | -- |
|  | Week 18 | 599 | 153 | 26 | 22.5 – 29.5 | 590 | 130 | 22 | 18.7 – 25.3 |
| RV1 arm | Week 6 | 299 | 17 | 5.7 | 3.1 – 8.3 | -- | -- | -- | -- |
|  | Week 18 | 305 | 96 | 31.5* | 26.3 – 36.7 | 299 | 80 | 26.8**^$^** | 21.4 – 31.4 |
| No RV1 arm | Week 6 | 291 | 10 | 3.4 | 1.3 – 5.5 | -- | -- | -- | -- |
|  | Week 18 | 294 | 57 | 19.4* | 14.9 – 23.9 | 291 | 50 | 17.2**^$^** | 12.7 – 21.3 |

^a^ seropositive defined as an anti-rotavirus IgA antibody titer ≥20 U/ml

^b^ seroconversion defined as < 20 U/ml rotavirus IgA at week 6 and ≥20 U/ml at week 18

*(*P* = 0.001) **^$^**(*P* = 0.005)

Table 6: Evaluation of variables associated with risk of rotavirus diarrhea and interaction of variables with RV1: Multivariable logistic regression best subsets analysis

| **Variable** | **Odds Ratio (95% CI)** | **Main effect** *P***-value** | **RV1 Interaction**  ***P*-value** |
| --- | --- | --- | --- |
| **Rotavirus Diarrhea in year 1** | | | |
| No RV1 arm (control) | 2.60 (1.78 – 3.80) | **4.7 × 10^–7^** | - |
| Zinc, 18 weeks (x 100ug/L) | 0.81 (0.70 – 0.93) | 0.002 | 0.476 |
| Monthly income (1000 Taka) | 1.02 (1.00 – 1.04) | 0.039 | 0.821 |
| Duration excl. breast feeding, 18 weeks | 0.97 (0.94 – 1.00) | 0.054 | 0.378 |
| Absence of water treatment | 1.47 (0.99 – 2.17) | 0.055 | 0.624 |
| Sex of child (male) | 1.36 (0.94 – 1.98) | 0.104 | 0.079 |
| WAZ, 10 weeks | 0.85 (0.69 – 1.04) | 0.108 | 0.999 |
| **Severe Rotavirus Diarrhea in year 1** | | | |
| No RV1 arm (control) | 3.18 (1.68 – 6.00) | 0.0001 | - |
| Monthly income (1000 Taka) | 1.03 (1.00 – 1.05) | 0.032 | 0.909 |

*P*-values from Likelihood Ratio Test

**Figure 2:** Impact of RV1 vaccination on Rotavirus Diarrhea in Vaccinated and Unvaccinated Bangladeshi children.
