# Supplementary material for: Impact of enterovirus and other enteric pathogens on oral polio and rotavirus vaccine performance in Bangladeshi infants
Source: Vaccine. 2016 Jun 8;34(27):3068–75. doi: 10.1016/j.vaccine.2016.04.080 (PMC4912219; doi:10.1016/j.vaccine.2016.04.080)
Supplement: Supplementary file 2 [file mmc2.pdf]

The Impact of Serum Zinc and Delayed Dosing on Rotavirus Gastroenteritis and Rotavirus Oral Vaccine Performance in Bangladeshi Infants

Oral Rotavirus Vaccine Underperformance

E. Ross Colgate, MPH. The University of Vermont Vaccine Testing Center and Unit of Infectious Diseases, Department of Medicine, College of Medicine, Burlington, VT, USA

Rashidul Haque, MD. The International Centre for Diarrhoeal Disease Research, Bangladesh (iccdr,b), Mohakhali, Dhaka, Bangladesh

Dorothy M. Dickson, MSc. The University of Vermont Vaccine Testing Center and Unit of Infectious Diseases, Department of Medicine, College of Medicine, Burlington, VT, USA

Marya P. Carmolli, BS. The University of Vermont Vaccine Testing Center and Unit of Infectious Diseases, Department of Medicine, College of Medicine, Burlington, VT, USA

Josyf C. Mychaleckyj, D.Phil. Department of Public Health Sciences and Center for Public Health Genomics, University of Virginia, VA, USA

Uma Nayak, Ph.D. Center for Public Health Genomics, University of Virginia, VA, USA

Firdausi Qadri, Ph.D. The International Centre for Diarrhoeal Disease Research, Bangladesh (iccdr,b), Mohakhali, Dhaka, Bangladesh

Masud Alam, MBBS. The International Centre for Diarrhoeal Disease Research, Bangladesh (iccdr,b), Mohakhali, Dhaka, Bangladesh

Mary Claire Walsh, MSc. The University of Vermont Vaccine Testing Center and Unit of Infectious Diseases, Department of Medicine, College of Medicine, Burlington, VT, USA

Sean A. Diehl, Ph.D. The University of Vermont Vaccine Testing Center and Unit of Infectious Diseases, Department of Medicine, College of Medicine, Burlington, VT, USA

K. Zaman, MD. The International Centre for Diarrhoeal Disease Research, Bangladesh (iccdr,b), Mohakhali, Dhaka, Bangladesh

William A. Petri, Jr., MD*. Division of Infectious Diseases and International Health, University of Virginia School of Medicine, Charlottesville, VA, USA

Beth D. Kirkpatrick, MD*. The University of Vermont Vaccine Testing Center and Unit of Infectious Diseases, Department of Medicine, University of Vermont College of Medicine, Burlington, VT, USA

*shared senior author

Corresponding Authors:
Beth D. Kirkpatrick, MD
University of Vermont Vaccine Testing Center and Unit of Infectious Diseases
University of Vermont College of Medicine
89 Beaumont Avenue

Burlington, VT 05405
Phone: 802 656 1121
Email:beth.kirkpatrick@uvm.edu

William A. Petri, Jr., MD.
Division of Infectious Diseases and International Health, University of Virginia School of Medicine, Charlottesville, VA, USA
Phone: (434) 924 5621
Email:wap3g@cms.mail.virginia.edu

Keywords

Rotavirus diarrhea

Zinc

Oral vaccine

Underperformance

Developing world

40-Word Summary

We examined oral rotavirus vaccine underperformance in a birth cohort of 700 children in Dhaka, Bangladesh. Vaccine efficacy was higher than expected, likely due to delayed dosing and active community-based diarrhea surveillance. Zinc was associated with protection against rotavirus diarrhea.

Abstract

Background: Rotavirus remains the world's leading cause of diarrheal death in children. Despite successes, oral rotavirus vaccines are less effective in developing countries. Reasons for vaccine underperformance remain unclear.

Methods: We performed a randomized, controlled trial of monovalent live-attenuated oral rotavirus vaccine (RV1) in Dhaka, Bangladesh to investigate vaccine underperformance. Infants were randomized 1:1 to receive RV1 or no RV1 using a delayed dosing schedule of 10 and 17 weeks. Intensive in-home diarrheal surveillance was performed and nutritional, socioeconomic, and immunologic variables were assessd. The primary outcome was ≥1 episode of rotavirus diarrhea (RVD).

Results: 700 healthy newborns were enrolled and followed for one year; 50% received RV1. Community incidence of rotavirus disease incidence was high, 38.3 cases/ 100 person years. Post-vaccination efficacy against severe RVD was higher than anticipated at 73.5% (95% CI 45.8 – 87.0), using delayed dosing. RV1 had no efficacy against all-cause diarrhea. Serum zinc (OR 0.81, p=0.002) and family income (OR 1.02, p=0.039) were associated with protection from RVD independent of vaccination. Zinc was not protective against non-rotavirus diarrhea. Nutritional status and vitamin A were not associated with RVD; duration of exclusive breastfeeding and water treatment were of borderline significance (*P*=0.05)

Conclusions: Intensive diarrhea surveillance and delayed vaccine dosing revealed an enhanced efficacy of RV1 against severe rotavirus diarrhea, despite a high incidence of rotavirus disease. Independent of the impact of vaccination, community wide-interventions, such as Zinc supplementation, may specifically decrease risk of rotavirus diarrhea.

Introduction

Rotavirus is the leading cause of child death from diarrhea. In 2008, prior to vaccine introduction, rotavirus diarrhea led to approximately 453,000 deaths, most in South Asia and Sub-Saharan Africa [1, 2]. Three oral live-attenuated rotavirus vaccines are now licensed: the 3-dose pentavalent human-bovine vaccine (RV5), the 2-dose monovalent G1P[8] vaccine (RV1), and the 3-dose human-bovine G9P[11] vaccine (116E). In phase III trials, RV5 and RV1 have robust efficacy of >85% in high-income countries, as measured by protection from either severe diarrhea or rotavirus diarrhea-related hospitalization following vaccination [3-5]. In contrast, efficacy is markedly lower in developing countries with the highest burden of rotavirus [5]. In multiple clinical trials, rotavirus vaccine efficacy ranges from 18% to 61% in Africa and Asia [6-10].

While lower rotavirus vaccine efficacy in developing countries is well-established, little is understood about the biologic basis of oral rotavirus vaccine underperformance. Previous efforts have postulated mechanisms related to the vaccine itself, including dosing schedule and inoculum, and factors impacting the child's ability to respond to vaccination [11, 12]. The latter includes factors that prevent the vaccine from replicating in the intestine or blunt infant immune responses, such as breast milk and maternal antibody interference, enteropathy, and enteric co-infections [13-17]. Nutritional status may also contribute. Specifically, dietary micronutrients such as zinc play an extensive role in host defense and gut health [18, 19].

In order to assess factors related to oral rotavirus vaccine underperformance, we enrolled a 700-child birth cohort in an urban slum of Dhaka, Bangladesh in the PROVIDE Study: a randomized, controlled trial (RCT) of the two-dose RV1 vaccine with a delayed dosing schedule at 10 and 17 weeks of age (compared to 6-14 weeks in previous studies) to minimize interference by maternal antibodies. With a primary outcome of any rotavirus diarrhea in the first year of life, we conducted intensive, biweekly home-based diarrhea surveillance with laboratory-confirmed rotavirus diarrhea to determine RV1 efficacy in this population, and assessed predictors of risk of rotavirus diarrhea and vaccine underperformance in the first year of life to determine targets for public health intervention and inform vaccine development efforts.

Methods

Study Design and Participants

We performed a 2x2 factorial randomized, open-label, controlled trial of live oral G1P[8] rotavirus vaccine (RV1) in a birth cohort of 700 children from the Mirpur urban slum of Dhaka, Bangladesh. Infants who met inclusion and exclusion criteria were enrolled in the first week of life. Detailed study methods including consenting procedures, eligibility criteria and protocol amendments are described elsewhere [20]. The study was approved by the research and ethical review boards of the icddr,b (Bangladesh), the University of Vermont, and the University of Virginia. Clinicaltrials.gov registration: NCT01375647.

Randomization and Masking

Children were randomized using permuted blocks with random block size selection (4 or 8). Children were randomly assigned to one of two treatment groups: 50% (n=350) to receive RV1 vaccine at weeks 10 and 17, and 50% no rotavirus vaccine. Sealed envelopes containing the random treatment group assignment and a unique study identification number (SID) were produced by the UVa Data Coordinating Center. SIDs with treatment group were assigned sequentially to each infant following enrollment. All clinical investigators and laboratories were masked to vaccine arm but not medical officers.

Procedures

The study was conducted from May 2011 through November 2013. Children were enrolled from birth to age seven days in the home by trained Field Research Assistants (FRAs), following comprehensive consenting procedures according to ICH GCP guidelines. Mothers were administered a baseline survey at enrollment to collect demographics, as well as household socio-economic and water and sanitation data.

There were ten protocol-driven clinic visits in the first year of life for anthropometry measurements, blood draws, and vaccinations. Children received the Bangladesh Expanded Program on Immunization (EPI) vaccines through the study clinic, including concomitant trivalent oral polio vaccine at week 10 for children in the RV1 dosing treatment arm. All acute illnesses were evaluated by medical officers, and RV1 dosing was delayed in children presenting with fever at scheduled vaccination visits. Breast feeding was not withheld. Vaccine cold chain was reviewed before administration. Children with severe malnutrition (WAZ < -3SD) were referred for specialized care.

Complete diarrhea surveillance was conducted throughout the first year of life [20]. FRAs visited households twice weekly to determine diarrheal episodes through a structured questionnaire, and one diarrheal stool sample was collected during each distinct episode. Mothers brought children into the clinic for further assessment and treatment of diarrheal illness. Diarrheal stool specimens were tested for rotavirus antigen by PosSpecT ELISA (Oxoid ltd., Hampshire, UK).

Blood specimens for immunogenicity and micronutrients were collected at weeks 6 and 18 using trace metal-free tubes. Plasma was evaluated for rotavirus specific IgA antibodies at weeks 6 and 18 as described [21]. Briefly, a capture EIA was performed using the rotavirus SA11 antigen; results were expressed as U/mL as determined by a positive control reference serum. Seropositive was defined as any specimen with rotavirus IgA ≥20 U/mL; seroconversion was defined as seropositivity following a seronegative result pre-vaccination. Vitamin D and retinol binding protein (RBP) levels were assessed using commercial ELISA kits (Immunodiagnostics Systems LTD, Tyne, United Kingdom and R&D Systems, Minneapolis, MN), and serum zinc was analyzed using fluorescence methods.

Outcomes

The primary outcome for efficacy analysis was one or more episodes of rotavirus diarrhea from birth to one year of age. Rotavirus diarrhea was defined as diarrhea positive for rotavirus antigen by ELISA. Diarrhea was defined as three or more abnormally loose stools in 24 hours; >72 hours separated distinct episodes. Severe diarrhea was defined as Vesikari score ≥11 [22]. Secondary outcomes were severe rotavirus diarrhea, all-cause diarrhea (diarrhea of any etiology) and all-cause severe diarrhea in the first year of life, and any and severe rotavirus diarrhea post-vaccination, from 18-52 weeks of age.

Statistical Analysis

The trial was designed with at least 90% power to detect 50% vaccine efficacy at $\alpha=0.05$, assuming rotavirus infection in 26% of non-vaccinated children by one year of age [15]. Primary analysis was by intention-to-treat (ITT): all randomized subjects were included regardless of whether they adhered to the protocol vaccine regimen or terminated study participation prior to one year. Secondary per-protocol (PP) analyses were also performed.

For dichotomous outcomes, proportions with Wilson 95% confidence intervals and absolute risk differences in vaccinated and unvaccinated groups were calculated [23, 24]. Vaccine efficacy was calculated using the standard formula: $(AR_{UNVAX} - AR_{VAX})/ (AR_{UNVAX} \times 100)$ with AR_{VAX}, attack rate in vaccinated and AR_{UNVAX}, attack rate in unvaccinated.

Logistic regression was used to analyze variables associated with risk of rotavirus diarrhea and severe rotavirus diarrhea. Considering the study size, thus limitation on outcome events, a limited pool of 12 explanatory variables was selected based on the literature and biologic plausibility. Univariate analyses investigated independent associations with rotavirus diarrhea. All 12 variables were then analyzed by best subsets multivariable logistic regression to determine the optimal model to explain risk of rotavirus diarrhea. Variables in the final model were chosen to minimize Mallows's Cp statistic and the Akaike Information Criterion (AIC) [25-27]. In the final model, vaccination arm (RV1 vs. no RV1) was tested with each of the other variables for significant interactions by examining the difference in deviance from the reduced model (Likelihood Ratio test). Analyses were performed using SAS 9.3 (Cary, North Carolina).

Results

Baseline

After a community-wide survey of the Mirpur area of Dhaka, Bangladesh, 700 mother-child pairs were consented and enrolled within seven days of birth (median age 5 days, range 1-7 days), per Figure 1. Children were randomized to receive the two-dose RV1 vaccine at 10 and 17 weeks of age or not. Population demographics are described elsewhere [20]. Baseline features by randomization arm are shown on **Table 1** and reflect the local population, including incidence of malnutrition, breast feeding, monthly income, and access to municipal water. There were no differences in baseline characteristics between the treatment arms. There were 92 drop outs followed for less than one year, 12% (n=43) in the RV1 arm and 14% (n=49) in the control arm, which did not differ between the trial arms ($P = 0.53$).

Incidence and Efficacy

Incidence of rotavirus diarrhea in unvaccinated children was 38.3 cases per 100 person-years, up to four times higher than incidence previously reported in developing countries, including Bangladesh (**Table 2**). In the primary analysis, vaccinated children vs. unvaccinated had significantly less rotavirus diarrhea (19.1 vs. 32.6%) and severe rotavirus diarrhea (4 vs 11.1%) in the first year of life, per ITT (**Table 3** and **Figure 2**). Overall vaccine efficacy was 41.2% (95%

CI: 23.6-54.8) against all rotavirus diarrhea and 64.1% (95% CI: 35.1-80.1) against severe rotavirus diarrhea. The number of children needed to treat (NNT) to prevent one case of rotavirus diarrhea, derived as the reciprocal of the absolute risk difference, was 8 (95% CI: 6-15). There were 2 confirmed cases of severe RVD between week 6 (first dose of the usual RV1 dosing regimen) and week 10 when the first dose of RV1 was given in PROVIDE.

To compare the efficacy estimate in PROVIDE to published trials, per-protocol analysis of efficacy post-vaccination (18-52 weeks) was also performed. Per protocol efficacy against all rotavirus diarrhea was 51% (95% CI: 33.8-63.7) and 73.5% (95% CI: 45.8-87) against severe rotavirus diarrhea (**Table 3**). Efficacy estimates against all-cause diarrhea suggest a lack of effect: 1.3% (95% CI: -4.8-7.1) for any case of diarrhea and 12.7% (95% CI: -7.5-29) for severe diarrhea (**Table 3**).

Factors Associated with Vaccine Performance

To evaluate factors for association with risk of rotavirus diarrhea and to ultimately evaluate the interaction of these variables with vaccine performance, univariate analysis of selected variables (**Table 4**) was initially performed by logistic regression. There were 588 children with no missing data for all explanatory variables included in this analysis.

Features significantly associated with increased risk of rotavirus diarrhea in the first year of life included, as anticipated, lack of rotavirus vaccine ($P < 0.001$, OR 2.34), as well as lack of water treatment in the household ($P = 0.037$, OR 1.47) (**Table 4**). Protective variables included serum zinc at week 18 ($P = 0.003$, OR 0.82) and exclusive breastfeeding duration ($P = 0.017$, OR 0.97) (**Table 4**). Not associated with risk for rotavirus diarrhea throughout the first year of life were nutritional status, income, and maternal education. Serum IgA at week 18 also was not associated with vaccine efficacy, although vaccinated children were significantly more likely to be seropositive and have seroconverted than unvaccinated children ($P = 0.001$ and 0.005 respectively, Chi-square test). That said, only 26.8 % of vaccinated children seroconverted between weeks 6 and 18 vs 17.2% in the unvaccinated arm (**Table 5**).

In multivariable best subsets analysis, variables which increased risk for rotavirus diarrhea included not receiving the rotavirus vaccine/control arm ($P < 0.001$) and lower household income (P= 0.039) (**Table 6**). Variables decreasing risk of rotavirus diarrhea in the first year of life included serum zinc at 18 weeks of life ($P = 0.002$). Notably, zinc level was not associated

with decreased risk from diarrhea due to other pathogens. Duration of exclusive breastfeeding and absence of water treatment were of borderline statistical significance (*P*=0.054 and 0.055) (**Table 6**).

Finally, to determine whether the explanatory variables modified the effect of the vaccine, tests of interaction between vaccine arm and each explanatory variable were performed. None of the interaction terms in the model were significant, suggesting that serum zinc levels and higher income were positively associated with protection from rotavirus diarrhea, but did not impact vaccine efficacy itself since these effects were independent of the vaccine.

Similar analysis was performed to assess variables associated with risk for severe rotavirus diarrhea, limited to variables significant in the multivariable analysis for the primary endpoint. In the logistic regression model, only absence of the rotavirus vaccine (*P* = 0.0001) and lower income (*P* = 0.032) were associated with increased risk of severe rotavirus diarrhea (**Table 6**).

Discussion

Using a clinical primary outcome of rotavirus diarrhea, we performed a controlled efficacy trial of oral rotavirus vaccine given at weeks 10 and 17 after birth. We demonstrate that in a highly rotavirus-endemic urban population in Bangladesh, the efficacy of RV1 vaccine to prevent all rotavirus diarrhea over the first year of life by intent-to-treat analysis is 41% (NNT = 8) and 64% (NNT = 14) against severe rotavirus diarrhea. This work confirms a substantial unmet need in the control of rotavirus disease.

Surprisingly, using post-vaccination per-protocol analysis, we found RV1 efficacy of 51% vs. all rotavirus diarrhea and 73.5% against severe disease, higher than previously seen in developing world settings [6, 7]. The differences may be attributable to our delayed dosing schedule, which may have minimized maternal antibody interference with vaccination. Although a previous study in Pakistan assessed differences in vaccine immunogenicity (serum rotavirus IgA) between different dosing regimens [17], to our knowledge PROVIDE is the only efficacy trial based on a clinical endpoint (rotavirus diarrhea) to evaluate delayed dosing (10 and 17 weeks); other efficacy trials have tested 2-3 doses in the 6-16 week window. Formally comparing the efficacy of different dosing schedules within the same study would more comprehensively elucidate the role of maternal antibody and other immunologic factors in vaccine performance. Although our data require confirmation, our intense community-based diarrhea surveillance may have

revealed an important benefit to vaccine efficacy following a modest delay in dosing. Since most rotavirus diarrhea in this population occurs after 3 months of age (**Figure 2)**, there appears to be minimal additional risk of severe rotavirus disease in the weeks between standard (age 6 weeks) and our delayed dosing at 10 weeks (two cases in PROVIDE).

In addition to its impact on rotavirus diarrhea, past studies have suggested rotavirus vaccine may prevent diarrhea from all etiologies [3, 6, 8], for which rotavirus often is a large percentage [28]. In contrast, we saw no impact of rotavirus vaccination on all-cause diarrhea, reiterating the importance and burden of other enteric pathogens affecting this impoverished urban population [28].

To determine associations with rotavirus vaccine efficacy, we analyzed nutritional, socio-economic, hygiene, and immunologic variables for association with rotavirus diarrhea. By univariate analysis: receipt of vaccine, serum zinc, duration of breast feeding, and home water treatment are associated with decreased risk of rotavirus diarrhea. By multivariable best-subsets analysis, however, only serum zinc, monthly family income, and rotavirus vaccination are strongly associated with protection from rotavirus diarrhea. Duration of exclusive breast feeding and absence of water treatment were of borderline statistical significance and may prove significant in a larger study. After performing individual tests of interaction with the vaccine arm, however, no variables modified the effect of the vaccine itself, suggesting that although improvement in these variables would decrease risk and overall burden of rotavirus diarrhea in both groups, response to vaccine itself may not be improved. Thus, significant improvements in control of rotavirus disease could be envisioned by improving the baseline health and conditions of all children including public health interventions of zinc supplementation and exclusive breast-feeding.

Serum zinc, in particular, was significantly associated with an overall decreased risk of rotavirus diarrhea (OR 0.81, $P = 0.002$) irrespective of rotavirus vaccination status. We estimate an increase of 10μg/dL in serum zinc levels would result in 28 fewer cases of rotavirus diarrhea (4% decrease) in the first year of life. This observation recalls the importance of zinc in intestinal epithelial repair and immunologic response mechanisms critical for mucosal protection [18, 29]. This finding may argue for zinc supplementation in young children beginning shortly after birth, as exclusive breast-feeding soon fails to provide adequate zinc [30]. Although our study was not powered to see an association with severe diarrhea, this observation deserves additional follow

up, particularly due to the ease of zinc administration. Notably, an association of serum zinc was not seen with diarrhea from causes other than rotavirus, and future work may determine if zinc supplementation may protect from specific pathogens. We also failed to find an association of malnutrition (HAZ, WAZ) and retinol binding protein levels (correlate of vitamin A) at the time of vaccination, with rotavirus diarrhea or vaccine failure.

Our findings regarding immunologic responses to vaccination raise concerns: less than one-third of children seroconverted (≥20 U/mL) following vaccination. The mean rotavirus-IgA titer was 12.2 U/mL in vaccinated children; markedly lower than in developed-country populations where levels often exceed 150 U/mL [31]. Low serum rotavirus-IgA levels after vaccination are consistent with observations in other low-income populations [31], but correlate poorly with vaccine efficacy, as reiterated by our best sub-sets results (no association).

Our work has limitations. Although we performed intense diarrhea surveillance, results may under-report the incidence of rotavirus diarrhea, particularly for short-duration diarrhea episodes for which specimens were not captured. We assumed these were negative for rotavirus, which may downwardly bias our vaccine efficacy estimates. Our trial results may be subject to the Hawthorne Effect in which children enrolled in the study receive high-standard primary care, potentially upwardly biasing our efficacy estimates. Additionally, the population size limited our best subsets analysis of final variables. Assessment of the impact of zinc on rotavirus diarrhea was limited to two time points (6 and 18 weeks of age).

While our results may be applicable to developing world populations, additional data and validation are necessary to fully understand the limitations of vaccine efficacy in developing countries and delineate a public health response. Recent work on the same cohort contributes an important part of this puzzle by teasing out the impact of markers of Environmental Enteric Dysfunction, including neopterin and plasma sCD14, on oral vaccine efficacy and immunogenicity [32]. Future work will focus on the impact of maternal antibodies and blood group antigens; the role of enteric co-pathogens at the time of vaccination; the effect of zinc supplementation and whether zinc has a pathogen-specific effect in protection from diarrheal disease; and the role of asymptomatic infection in rotavirus vaccine underperformance. Our work reiterates the importance of an effective oral rotavirus vaccine in highly endemic settings and the importance of understanding and resolving issues of immune un-responsiveness.

Notes

Funding. This work was supported by the Bill and Melinda Gates Foundation, Seattle, Washington, USA

Acknowledgements. We thank the PROVIDE Study families for their willingness to participate. We acknowledge all PROVIDE Study staff for their hard work and dedication; Dr. R. Bradley Sack, Independent Medical Monitor; and the PROVIDE Study External Advisory Board (Drs. Zulfiqar Bhutta, Roger Glass, Brian Kelsall, Marian Neutra, Walt Ornstein, Marc Pallansch, Duncan Steele).

References

1. Black RE, Cousens S, Johnson HL, et al. Global, regional, and national causes of child mortality in 2008: a systematic analysis. Lancet **2010**; 375: 1969-87.

2. Tate JE, Burton AH, Boschi-Pinto C, et al. 2008 estimate of worldwide rotavirus-associated mortality in children younger than 5 years before the introduction of universal rotavirus vaccination programmes: a systematic review and meta-analysis. The Lancet infectious diseases **2012**; 12(2): 136-41.

3. Ruiz-Palacios GM, Perez-Schael I, Velazquez FR, et al. Safety and efficacy of an attenuated vaccine against severe rotavirus gastroenteritis. The New England journal of medicine **2006**; 354(1): 11-22.

4. Vesikari T, Matson DO, Dennehy P, et al. Safety and efficacy of a pentavalent human-bovine (WC3) reassortant rotavirus vaccine. The New England journal of medicine **2006**; 354(1): 23-33.

5. Nelson EA, Glass RI. Rotavirus: realising the potential of a promising vaccine. Lancet **2010**; 376(9741): 568-70.

6. Zaman K, Dang DA, Victor JC, et al. Efficacy of pentavalent rotavirus vaccine against severe rotavirus gastroenteritis in infants in developing countries in Asia: a randomised, double-blind, placebo-controlled trial. Lancet **2010**; 376(9741): 615-23.

7. Armah GE, Sow SO, Breiman RF, et al. Efficacy of pentavalent rotavirus vaccine against severe rotavirus gastroenteritis in infants in developing countries in sub-Saharan Africa: a randomised, double-blind, placebo-controlled trial. Lancet **2010**; 376(9741): 606-14.

8. Cunliffe NA, Witte D, Ngwira BM, et al. Efficacy of human rotavirus vaccine against severe gastroenteritis in Malawian children in the first two years of life: a randomized, double-blind, placebo controlled trial. Vaccine **2012**; 30 Suppl 1: A36-43.

9. Bhandari N, Rongsen-Chandola T, Bavdekar A, et al. Efficacy of a monovalent human-bovine (116E) rotavirus vaccine in Indian children in the second year of life. Vaccine **2014**; 32 Suppl 1: A110-6.

10. Madhi SA, Cunliffe NA, Steele D, et al. Effect of human rotavirus vaccine on severe diarrhea in African infants. The New England journal of medicine **2010**; 362(4): 289-98.

11. Angel J, Franco MA, Greenberg HB. Rotavirus vaccines: recent developments and future considerations. Nature reviews Microbiology **2007**; 5(7): 529-39.

12. Neuzil KM, Zaman K, Victor JC. A proposed framework for evaluating and comparing efficacy estimates in clinical trials of new rotavirus vaccines. Vaccine **2014**; 32s1: A179-a84.

13. Clemens J, Rao M, Ahmed F, et al. Breast-feeding and the risk of life-threatening rotavirus diarrhea: prevention or postponement? Pediatrics **1993**; 92(5): 680-5.

14. Rongsen-Chandola T, Strand TA, Goyal N, et al. Effect of withholding breastfeeding on the immune response to a live oral rotavirus vaccine in North Indian infants. Vaccine **2014**; 32 Suppl 1: A134-9.

15. Mondal D, Minak J, Alam M, et al. Contribution of enteric infection, altered intestinal barrier function, and maternal malnutrition to infant malnutrition in Bangladesh. Clinical infectious diseases : an official publication of the Infectious Diseases Society of America **2012**; 54(2): 185-92.

16. Babji S, Kang G. Rotavirus vaccination in developing countries. Current opinion in virology **2012**; 2(4): 443-8.

17. Ali SA, Kazi AM, Cortese MM, et al. Impact of different dosing schedules on the immunogenicity of the human rotavirus vaccine in infants in Pakistan: a randomized trial. The Journal of infectious diseases **2014**; 210(11): 1772-9.

18. Shankar AH, Prasad AS. Zinc and immune function: the biological basis of altered resistance to infection. The American journal of clinical nutrition **1998**; 68(2 Suppl): 447s-63s.

19. Brown KH, Rivera JA, Bhutta Z, et al. International Zinc Nutrition Consultative Group (IZiNCG) technical document #1. Assessment of the risk of zinc deficiency in populations and options for its control. Food and nutrition bulletin **2004**; 25(1 Suppl 2): S99-203.

20. Kirkpatrick BD, Colgate ER, Mychaleckyj JC, et al. The “Performance of Rotavirus and Oral Polio Vaccines In Developing Countries” (PROVIDE) Study: Description of Methods of an Interventional Study Designed to Explore Complex Biologic Problems. American Journal of Tropical Medicine and Hygeine **2015**; in press.

21. Azim T, Ahmad SM, Sefat EK, et al. Immune response of children who develop persistent diarrhea following rotavirus infection. Clinical and diagnostic laboratory immunology **1999**; 6(5): 690-5.

22. Ruuska T, Vesikari T. Rotavirus disease in Finnish children: use of numerical scores for clinical severity of diarrhoeal episodes. Scandinavian journal of infectious diseases **1990**; 22(3): 259-67.

23. Wilson EB. Probable Inference, the Law of Succession, and Statistical Inference. Journal of the American Statistical Association **1927**; (22): 209-12.

24. Newcombe RG. Interval estimation for the difference between independent proportions: comparison of eleven methods. Stat Med **1998**; 17(8): 873-90.

25. Akaike H. Information theory and an extension of the maximum likelihood principle. In: 2nd International Symposium on Information Theory. Tsahkadsor, Armenia, USSR: Akadémiai Kiadó, 1971:267-81.

26. Mallows CL. Some Comments on C_p. Technometrics **1973**; 15(4): 661-75.

27. Burnham KP, Anderson DR. Model Selection and Multimodel Inference: A Practical Information-Theoretic Approach. 2nd ed: Springer-Verlag, **2002**.

28. Kotloff KL, Nataro JP, Blackwelder WC, et al. Burden and aetiology of diarrhoeal disease in infants and young children in developing countries (the Global Enteric Multicenter Study, GEMS): a prospective, case-control study. Lancet **2013**; 382(9888): 209-22.

29. Haase H, Rink L. Functional significance of zinc-related signaling pathways in immune cells. Annual review of nutrition **2009**; 29: 133-52.

30. Brown KH, Engle-Stone R, Krebs NF, Peerson JM. Dietary intervention strategies to enhance zinc nutrition: promotion and support of breastfeeding for infants and young children. Food and nutrition bulletin **2009**; 30(1 Suppl): S144-71.

31. Patel M, Glass RI, Jiang B, Santosham M, Lopman B, Parashar U. A systematic review of anti-rotavirus serum IgA antibody titer as a potential correlate of rotavirus vaccine efficacy. The Journal of infectious diseases **2013**; 208(2): 284-94.

32. Naylor C, Lu M, Haque R, et al. Environmental Enteropathy, Oral Vaccine Failure and Growth Faltering in Infants in Bangladesh. EBioMedicine **2015**; 2(11): 1759-66.
